# Supplementary material for: Mullite-Fibers-Reinforced Bagasse Cellulose Aerogels with Excellent Mechanical, Flame Retardant, and Thermal Insulation Properties
Source: Materials (Basel). 2024 Jul 28;17(15):3737. doi: 10.3390/ma17153737 (PMC11312846; doi:10.3390/ma17153737)
Supplement: Supplementary file 1 [file materials-17-03737-s001.zip › materials-3120869-supplementary.pdf]

Supporting Information:

Mullite-Fibers-Reinforced Bagasse Cellulose Aerogels with Excellent Mechanical, Flame Retardant, and Thermal Insulation Properties

Shuang Wang [1,†], Miao Sun [1,†], Junyi Lv [1], Jianming Gu [1], Qing Xu [1], Yage Li [1], Xin Zhang [1], Hongjuan Duan [1,*] and Shaoping Li [2]

[1] The State Key Laboratory of Refractories and Metallurgy, Wuhan University of Science and Technology, Wuhan 430081, China; shuangwang@wust.edu.cn (S.W.); miao740526@163.com (M.S.); lvjunyi@wust.edu.cn (J.L.); gjm15503921886@163.com (J.G.); xuqing99@yahoo.com (Q.X.); liyg@zjweu.edu.cn (Y.L.); 17634809427@163.com (X.Z.)

[2] Hubei Three Gorges Laboratory, Yichang 443007, China; lisp@xingfagroup.com

* Correspondence: duanhongjuan@wust.edu.cn

† These authors contributed equally to this work.

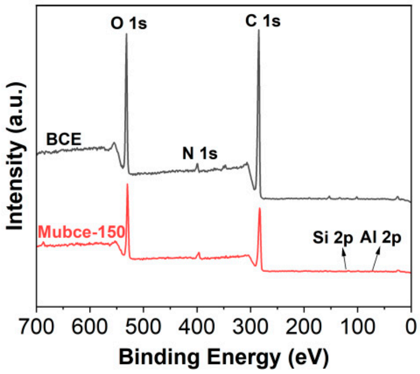

Figure S1. XPS spectra of BCE and Mubce-150 aerogels.

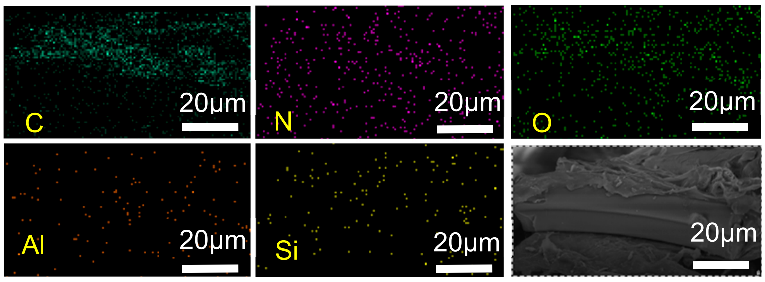

Figure S2. The EDS spectra of the selected area of Mubce-150 aerogels.

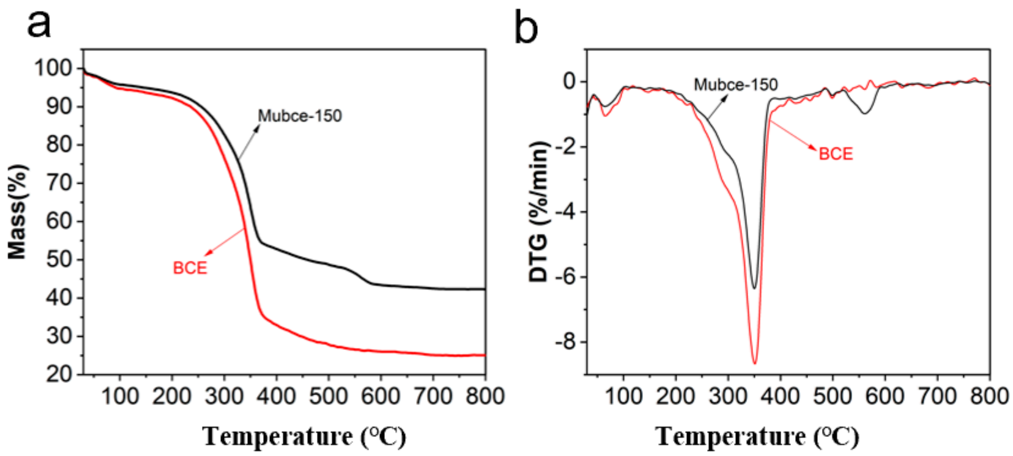

Figure S3. TGA (a) and DTG (b) of BCE and Mubce-150 aerogels (under argon atmosphere).

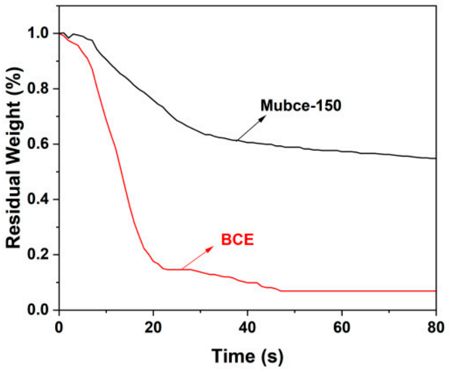

Figure S4. Residual weight versus time curves of BCE and Mubce-150 aerogels.

Table S1. Performance comparison of biomass aerogels in previously published literatures

Raw materials	Porosity (%)	Compressive strength (kPa)	Thermal conductivity(W /m·K)	Recovery rate after 100 cycles (%)	Stress retention after 100 cycles (%)	Ref.
Cotton fibers/phytic acid	95.6	5 (10%)	0.0352	91.3(50%)	-	1
Cellulose nanofiber /chitosan	98.0	60 (60%)	0.036	90 (60%)	84.7 (60%)	2
Hemp microfibers/silane derivative	99.9	30 (80%)	0.0215	78.0(40%)	90.0 (40%)	3
Natural down-fiber /silk fibroin	97.6	146(60%)	0.034	82.9(60%)	-	4
Bacterial cellulose/Methyltrimethoxysilane	99.1	68(80%)	0.0273	-	-	5
Hardwood pulp/ deep eutectic solvent	-	5(80%)	-	80.5(80%)	90.0 (50%)	6
Mullite Fiber / Bagasse Cellulose	93.2	272（60%）	0.0276	81.6(40%)	92.7%(40%)	This work

Table S2. Comparison of thermal insulation properties of present aerogels with previously reported results

Materials	Thickness(mm)	Heat temperature/Materials surface temperature (℃)	Ref.
Cotton fibers/Phytic acid	1.0	150/71.8	1
Natural down-fiber reinforced silk fibroin	20.0	160/42.2	4
SiO_2/cellulose nanofiber	21.2	280/53.9	7
Polybenzoxazine/cellulose nanofiber	18.3	300/59.3	8
Polyvinyl alcohol and cellulose nanofibrils	5.0	90/6.6	9
Silica aerogel composite	6.0	300/90.5	10
Mullite Fiber / Bagasse Cellulose	22.0	160/33.8	This work

Table S3. Thermal stability of BCE and Mubce-150 aerogels.

Sample	$T_{-10\%}$ (℃)	$T_{-50\%}$ (℃)	T_{-max} (℃)	Residue (%)
BCE aerogels	235.5	349.5	351.5	25.1
Mubce-150 aerogels	255.4	458.4	350.4	42.3

Video S1. Video of the Mubce aerogels compressed in liquid nitrogen

Video S1.mp4

Video S2. Video of the BCE aerogels continuously burned by a butane spray gun

Video S2 BCE.mp4

Video S3. Video of the Mubce aerogels continuously burned by a butane spray gun

Video S3 Mubce.mp4

References

1. Wei, L.; Sun, L.; Zhao, H.; Lu, J.; Liu, L.; Yao, J. Aggregation-induced microfilaments enabled cotton cellulose aerogels with highly compressive and fatigue resistance, greatly thermal insulation and fireproofing. *Ind Crop Prod*. **2023**, *206*, 117666.
2. Zhang, M.; Jiang, S.; Han, F.; Li, M.; Wang, N.; Liu, L. Anisotropic cellulose nanofiber/chitosan aerogel with thermal management and oil absorption properties. *Carbohyd Polym*. **2021**, *264*, 118033.
3. Zhu, J.; Zhu, Y.; Ye, Y.; Qiu, Z.; Zhang, Y.; Yu, Z.; Sun, Xia.; Bressler, D. C.; Jiang, Feng. Super elastic and Ultralight Aerogel Assembled from Hemp Microfibers. *Adv Funct Mater*. **2023**, 33.
4. Shi, Y.; Miao, Y.; Li, L.; Li, W.; Zheng, X.; Zhao, J.; Liu, Z. Efficient thermal insulation through all-biomass silk fibroin composite aerogel 3D-reinforced by natural down-fiber. *Mater Today Phys*. **2024**, *40,* 101323.
5. Ke, W.; Ge, F.; Shi, X.; Zhang, Y.; Wu, T.; Zhu, X.; Cheng, Y.; Shi, Y.; Wang, Z.; Yuan, L.; Yan, Y. Superelastic and super flexible cellulose aerogels for thermal insulation and oil/water separation. *Int J Biol Macromol*. **2024**, 129245.
6. Ma, X.; Zhou, S.; Li, J.; Xie, F.; Yang, H.; Wang, C.; Fahlman, B. D.; Li, W. Natural microfibrils/regenerated cellulose-based carbon aerogel for highly efficient oil/water separation. *J Hazard Mater*. **2023**, *454*, 131397.
7. Long, X.; Wei, X.; Hu, M.; Yu, J.; Wang, S.; Zhou, L.; Liao, J. Anisotropic and high-strength SiO_2/cellulose nanofiber composite aerogel with thermal superinsulation and super hydrophobicity. *Ceram Int.* **2023**, *49*, 28621-28628.
8. Long, X.; Huang, P.; Wei, X.; Yu, J.; Wang, S.; Liao, J. Ultra-high strength, highly deformable and superhydrophobic polybenzoxazine@cellulose nanofiber composite aerogel for thermal insulation. *Compos Part A-Appl S*. **2023**, *175,* 107771.
9. Ding, M.; Ma, W.; Liu, P.; Yang, J.; Lan, K.; Xu, D. Creating aligned porous structure with cobweb-like cellulose nanofibrils in MXene composite aerogel for solar-thermal desalination and humidity response. *Chem Eng J.* **2023**, *459*, 141604.
10. Yu, D.; Liu, M.; Xu, F.; Kong, Y.; Shen, X. Structure tailoring and thermal performances of water glass-derived silica aerogel composite with high specific surface area and enhanced thermal stability. *J Non-Cryst Solids*. **2024**, *630*, 122889.
